# Literary Gossip and Record

**Published:** 1862-01

**Authors:** 


### ART. XI.—LITERARY GOSSIP AND RECORD.

A SINGULARLY interesting medical discussion of a disputed point of history, entitled *An Enquiry into the Circumstances of the Death of King Charles the Second*, by Dr. Norman Chevers, has been published in the last (XIV.) number of the *Indian Annals of Medical Science.*

By the majority of recent historical authorities it is considered that Charles II. died from apoplexy; by several contemporary writers it was suspected or asserted that he was poisoned. No medical man who reads the narrative of the case, Dr. Chevers is of opinion, will assent to the conclusion that the disease which proved fatal to the King was apoplexy; and notwithstanding that the historical foundation for the suspicion of foul play by poison is strong, medical grounds for this suspicion are entirely wanting.

Lord Macaulay has written that—"It should seem that no transactions in history ought to be more accurately known to us than those which took place round the deathbed of Charles the Second. We have several relations written by persons who, though not themselves eye-witnesses, had the best opportunities of obtaining information from eye-witnesses. Yet, whoever attempts to digest this vast mass of materials into a consistent narrative will find the task a difficult one. Indeed, James and his wife, when they told the story to the nuns of Chaillot, could not agree as to some circumstances. The Queen said that after Charles had received the last sacrament, the Protestant bishops renewed their exhortations: the King said that nothing of the kind took place. 'Surely,' said the Queen, 'you told me so yourself.' 'It is impossible that I could have told you so,' said the King, 'for nothing of the sort happened.'"

Since this was written, much important information has been collected bearing upon this event; and Dr. Chevers has from the different sources now accessible constructed a compacted and satisfactory account of the last illness of Charles II., enabling us to form a clear notion of its nature.

No abstract can do justice to or convey a correct notion of the great interest of Dr. Chevers's paper, but we shall endeavour to note a few points which may serve to indicate the reasons for the conclusion to which he ultimately comes.

The King appears to have been conscious of failing health for some considerable time prior to his last illness, and latterly he began to suffer from attacks of intermittent fever, the disease to which James I. and Cromwell fell victims. "His palaces at Whitehall and Windsor must have been infested with malarious exhalations from Lambeth Marsh and Eton flats; but he, in all probability, owed this disease mainly to his habits of feeding the ducks in the straight canal which then ornamented St. James's Park, and of fishing in the Itchin and in the Thames at Datchet." He too, for some time, complained that Windsor did not agree with him; and there is reason to believe that subsequent to his becoming subject to ague, he was alive to the precariousness of his existence, and, to a certain extent, anticipated the fatal result. "Having employed Sir Christopher Wren to build him a new palace at Winchester, the architect insisting that the building could not be creditably completed in less than two years, though possibly it might be finished, after a fashion, in one,—'If it be possible,' said Charles, 'let it be completed in that time: a year is a long period in my life.' He died a few weeks afterwards."

The first "fit" which the King suffered from occurred at a full levee, when he suddenly fell back in his chair with the exclamation of a dying man. Dr. Welwood, who was physician, for Scotland, to William III., but did not attend King Charles in his last illness, was told by an eyewitness that the King had been "suddenly surprized with *a fit, accompanied with violent convulsions of the body and contortions of the face, which lasted for some moments;*" and when a priest then present offered assistance, the King told the priest not to be afraid, for he had been "*troubled with the like before.*" Dr. Welwood also says: "It is known he (Charles) had been once or twice attacked before with fits that much resembled those of which he afterwards died. And yet, as the manner of them is told, *they look rather to have been convulsive motions than an apoplexy, seeing they were attended with violent contortions of his face, and convulsions of his whole body and limbs.*" Dr. Welwood conceives that a cause might be found for these attacks in the drying-up of a long-standing and profuse issue in the leg to which the King was subject, and which he had caused to be dried up against the advice of his physicians.

The King's fatal illness commenced on the morning of Monday, the 2nd February, 1685, after he had passed a feverish and restless night. On rising, it was noticed that his aspect was manifestly altered (Welwood says, "a ghastliness and paleness in his looks"); and before his dressing was completed, he was seized suddenly with a "fit." An official medical history of Charles's last illness says:—

February 2nd, 1685.—"Ad octavam præcisè horam Rex serenissimus, Carolus II., lecto recens relicto dum in cubiculo leniter inambulabat inordinatum querdan in cerebro sensit motum, cui mox aphonia motusque convulsivi vehementiores succedebant. Aderant fortè tunc ex Medicis Regiis omnino duo, qui, ut tanto Regum optimi periculo maturè prospicerent, venam ei in brachio dextro aperuerunt, sanguinisque eduxerunt uncias circiter sedecim."

After coming out of the fit, Welwood says, that the King suffered racking pain in the stomach. The Draycot MS. tells us that after he had come about, "he was very sensible, and told Dr. Short, but now, he could not speak, and asked what ailed him." From the same MS. we learn that in addition to the bleeding, "a warming-pan of coals was applied to the head; the back, arms, and thighs were blistered, and a puke was given."

The progress of the case on Tuesday and Wednesday, the 3rd and 4th of February, is not (in the absence of this portion of the official medical report) very clearly made out; but in the night following the first attack, according to the Draycot MS., the King "was taken with something like a return, between eleven and one, but it passed easily." Further, we learn from the same source that "*Since this, every night about the hours of twelve or one, he found an alteration, something of cold sweat, and some shivering.*" He was let blood from the jugulars on Wednesday. This would have been done on the first attack, "*but the convulsions were so strong and sudden they could not.*" He was also purged. On Wednesday or Thursday morning, the physicians, or the King himself, discovered that he was suffering from intermitting fever, and Jesuit's powder was given to him four times. On Thursday, the 5th, a second fit returned. "At eleven o'clock on Thursday night he asked the hour; and when they told him, he answered, 'Then half-an-hour after twelve I shall depart.' '(This presentiment,' says Dr. Chevers, 'is worthy of remark: we have seen that, every night at about midnight, there had been feverish exacerbation; the King evidently apprehended this, and believed that, exhausted as he was, the next paroxysm would carry him off.)' "

Some paroxysms of pain appear to have occurred during the night. "About six in the morning of Friday, the 6th, he complained of pain in the side, accompanied with difficulty of breathing, to remove which eight ounces of blood were taken from his arm. The Draycot MS. says, "he was then let blood by order of the Council, though the physicians despaired of life." He was dosed also with Raleigh's Antidote or Cordial. At half-past eight he spoke with difficulty, and a little after noon he died, "as peaceful as a lamb," says the author of the Draycot letter, "and had his sense, though not his speech, to the very last."

The medical report of the autopsy states—

"1. In cerebri cortice venæ et arteriæ super modum repletæ. 2. Cerebri tum ventriculi omnes serosâ quâdam materiâ inundati, tum ipsa substantia consimili humore haud leviter imbuta. 3. Thoraci dextri lateris pulmones pluræ tenaciter adhærentes sinistra vero plane liberi, quemadmodum ex naturæ instituto in sanis esse solet. 4. Pulmonum substantia neutiquam culpanda quidem sed sanguine referta. 5. Cor amplum firmumque, et in omnibus rectissimè formatum. 6. In infimo [intimo ?] ventre nihil præter naturale, nisi quod hepatis color ad lividitatem inclinaret, forte a sanguinis inibi restitantis pleonasmo, quo renes et lien cernebantur suffarcinati."

Dr. Chevers states that the whole of the recorded symptoms point to the conclusion that the disease from which Charles died was low intermittent or remittent fever with convulsions, the character of which was more or less epileptiform. He had long looked upon these convulsive fits as being the eclampsia which not unfrequently attends disease of the kidneys, the commonest effect of intemperate habits, and resulting in uræmia. This may, possibly, have been the case, the King's mode of living, and the condition of the kidneys—*sanguine suffarcinati*—justifying the suspicion. The presence of a gouty state of the system, and the recent suppression of the discharge from an issue in the leg, would add to the gravity of the case. He was indebted to Dr. Arthur Payne for the first hint as to what he considers the true explanation of the case; an explanation derived from the study of malarious fevers with convulsions in India.

A highly suggestive paper by Dr. Payne, on Epilepsy as a result of malarious infection, is contained in the same number of the *Indian Annals* in which Dr. Chevers's paper appears.

